# Protein Alterations in Cardiac Ischemia/Reperfusion Revealed by Spatial-Omics

**DOI:** 10.3390/ijms232213847

**Published:** 2022-11-10

**Authors:** Stephanie T. P. Mezger, Alma M. A. Mingels, Matthieu Soulié, Carine J. Peutz-Kootstra, Otto Bekers, Paul Mulder, Ron M. A. Heeren, Berta Cillero-Pastor

**Affiliations:** 1 Maastricht MultiModal Molecular Imaging (M4i) Institute, Division of Imaging Mass Spectrometry, Maastricht University, Universiteitssingel 50, 6229 ER Maastricht, The Netherlands; 2 Central Diagnostic Laboratory, Maastricht University Medical Center, P.O. Box 5800, 6202 AZ Maastricht, The Netherlands; 3 CARIM School for Cardiovascular Diseases, Maastricht University, Universiteitssingel 50, 6229 ER Maastricht, The Netherlands; 4 Normandie Université, UNIROUEN, Inserm U1096, Endothelium, Valvulopathy & Heart Failure, 76183 Rouen, France; 5 Pathologie Friesland, 8901 EN Leeuwarden, The Netherlands; 6 MERLN Institute for Technology-Inspired Regenerative Medicine, cBITE—Cell Biology-Inspired Tissue Engineering Department, Maastricht University, Universiteitssingel 40, 6229 ER Maastricht, The Netherlands

**Keywords:** myocardial infarction, ischemia/reperfusion injury, proteins, MALDI-MSI, LMD, spatial-omics

## Abstract

Myocardial infarction is the most common cause of death worldwide. An understanding of the alterations in protein pathways is needed in order to develop strategies that minimize myocardial damage. To identify the protein signature of cardiac ischemia/reperfusion (I/R) injury in rats, we combined, for the first time, protein matrix-assisted laser desorption/ionization mass spectrometry imaging (MALDI-MSI) and label-free proteomics on the same tissue section placed on a conductive slide. Wistar rats were subjected to I/R surgery and sacrificed after 24 h. Protein MALDI-MSI data revealed ischemia specific regions, and distinct profiles for the infarct core and border. Firstly, the infarct core, compared to histologically unaffected tissue, showed a significant downregulation of cardiac biomarkers, while an upregulation was seen for coagulation and immune response proteins. Interestingly, within the infarct tissue, alterations in the cytoskeleton reorganization and inflammation were found. This work demonstrates that a single tissue section can be used for protein-based spatial-omics, combining MALDI-MSI and label-free proteomics. Our workflow offers a new methodology to investigate the mechanisms of cardiac I/R injury at the protein level for new strategies to minimize damage after MI.

## 1. Introduction

Myocardial infarction (MI) is the most common cause of cardiovascular death and is a result of the blockage of coronary arteries leading to a reduced blood flow in the underlying cardiac tissue. Although early restoration of the blood flow is essential, by thrombolytic therapy or invasive procedures, this sudden reperfusion can cause additional myocardial injury, the so-called ischemia/reperfusion (I/R) injury [1,2]. After an ischemic event the heart can be classified in infarct (core), peri-infarct (or border) and remote myocardial regions, where complex processes take place, including structural changes and pathological processes, such as oxidative stress, activation of cell death, inflammation, and eventually remodeling [3,4,5,6].

The cardiac proteome has been already studied to obtain knowledge of the molecular factors involved in these pathological processes. For instance, different infarct models provided information on protein changes from early to late timepoints after reperfusion, mostly using tissue extracts [7,8,9]. Remote and peri-infarct areas have altered mechanical function, based on cardiac magnetic resonance studies [10], however only a few proteomic studies analyze these different regions separately [7,11,12].

In contrast to the proteomics approaches mentioned above, tissue analyses using matrix-assisted laser desorption/ionization mass spectrometry imaging (MALDI-MSI) provide molecular spatial information. MALDI-MSI has been previously applied to cardiovascular diseases [13] and revealed spatial changes at all molecular levels, from metabolites to proteins. Metabolites have been examined in an acute infarct mouse model, revealing distinct metabolic responses in the infarct core, border and remote regions by using this technology [14]. A more recent study evaluated metabolic changes in the first 4 h of ischemia, detecting significant changes already after 15 min while the myocardium appeared normal in histology [15]. At the lipid level, lysophospholipids were found to be increased while intact phospholipids were decreased in the infarct region 24 h after MI in rodent models [16,17]. These results suggest the increased activity of phospholipases, such as phospholipase A_2_, which is associated with inflammation. Lastly, proteins in MI have also been studied, but mostly as peptides after on-tissue digestion [18,19]. These studies indicated an increase in remodeling in the remote tissue, inflammatory response in the border region, and mitochondrial and metabolic enzymes in the infarct tissue.

The limited number of studies studying on-tissue intact protein distributions by MSI, indicate the challenge in the field of protein imaging. Here, the *m*/*z* alone does not provide sufficient information for confident protein identification. In addition, proteome coverage remains limited to the most abundant proteins due to the dynamic range. Protocols to overcome these challenges include on-tissue digestion or the use of additional separating techniques, such as ion mobility or liquid chromatography (LC), in general using tissue homogenates. Recent developments in the field of spatial-omics showed the combination of MALDI-MSI, laser capture microdissection (LMD) and proteomics [20,21]. We previously showed the use of this workflow on single conductive slides [22], and demonstrated that the protein content could be analyzed after lipid or metabolite MSI.

In the present proof-of-concept study, a (protein-based) spatial-omics approach was applied for the in-depth assessment of pathophysiological alterations in a cardiac I/R rat model by combining MALDI-MSI, LMD and LC-MS/MS on the same tissue section. This state-of-the-art approach allowed the identification of changes in protein content and the investigation of pathways involved in I/R injury, providing a new workflow for the development of strategies to minimize myocardial damage after MI.

## 2. Results

### 2.1. Cardiac Troponin as a Biomarker for Ischemic Heart Injury

**Figure 1:**
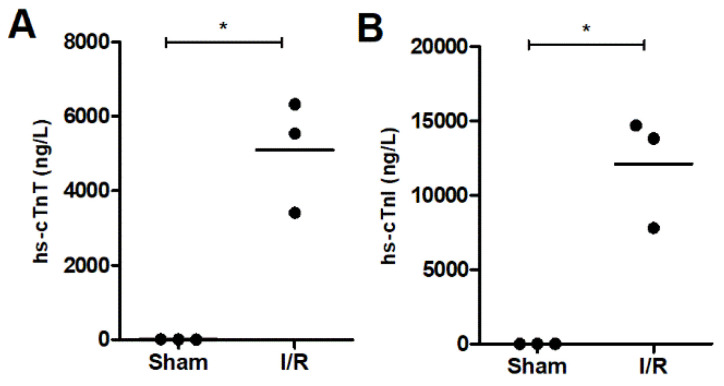
Cardiac troponin concentrations in circulating blood ((**A**), hs-cTnT and (**B**), hs-cTnI) for sham and I/R rats illustrating significant heart injury in the I/R rats. The mean is represented by the line and * indicates *p*-value ≤ 0.005.

The evaluation of ischemic heart injury was evaluated based on the concentrations of hs-cTnT and hs-cTnI in circulating blood (Figure 1). For the sham rats, concentrations were in the normal range [23,24], with a mean ± SD of 12.0 ± 2.9 ng/L for hs-cTnT and 0.5 ± 0.49 ng/L for hs-cTnI while in the I/R rats concentrations were significantly (*p* ≤ 0.005) elevated up to 5093 ± 1513 ng/L and 12,091 ± 3734 ng/L, respectively.

### 2.2. Spatial Distribution of Proteins in the I/R Heart

**Figure 2:**
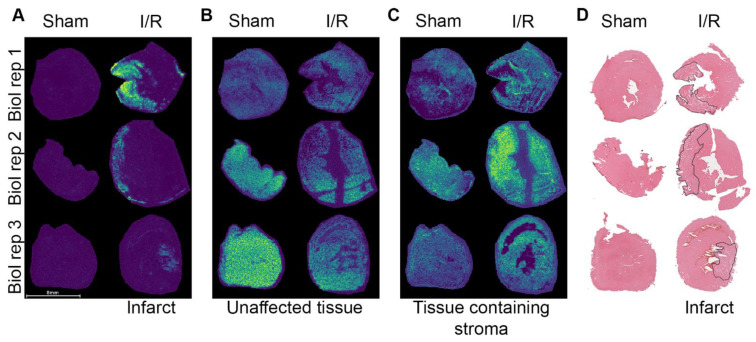
PLSA analysis revealed distinct regions within the I/R heart. These regions were identified as infarct (**A**), unaffected tissue (**B**) and tissue containing interstitial stroma (**C**). The infarct region is annotated in H&E-stained consecutive sections and represented in black (**D**, detailed annotations are depicted in Figure S4). Biol rep = biological replicate.

Both sham and I/R hearts were analyzed using MALDI-MSI to study the protein spatial distribution and to identify *m*/*z* values specific for distinctive regions within the heart after I/R injury (Figure S2). An unsupervised multivariate probabilistic latent semantic analysis (pLSA, using five components, Figure S3) showed three components which represented distinct regions in the tissue. These were identified as the infarct region (Figure 2A), unaffected tissue (Figure 2B) and tissue with interstitial stroma (Figure 2C), respectively. The assignment of these regions was confirmed by histological annotation of a consecutive section by a blinded trained pathologist (Figure 2D and Figure S4).

Furthermore, based on the distinctive regions obtained from the pLSA, ROC analyses were performed to look for region-specific *m*/*z* values (Table S1). This analysis revealed 11 *m*/*z* species-specific for the infarct region (AUC ≥ 0.8). For example, *m*/*z* 3332.7 and *m*/*z* 10150.4 (AUC 0.939 and AUC 0.935, respectively, Figure S5A,B) both showed a high intensity in the infarct area. Within the unaffected tissue 43 *m*/*z* species were distinctive (AUC < 0.2), such as *m*/*z* 17122.4 and *m*/*z* 8125.9 (AUC 0.097 and AUC 0.054, respectively, in Figure S5C,D). The comparison between the unaffected tissue in I/R (remote) versus sham hearts gave five unique *m*/*z* values (AUC ≥ 0.8), with a higher intensity in the I/R hearts.

**Figure 3:**
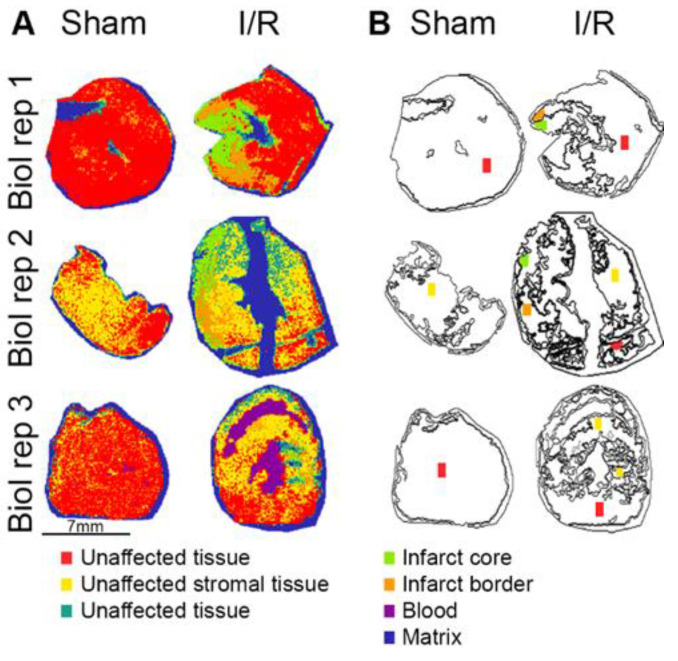
Segmentation analysis of sham and I/R hearts divided the tissue over seven clusters, separating infarct, unaffected tissue, blood and matrix. (**A**) The segmentation results showed a representation of the infarct core by the green and infarct border by orange clusters, while the red, yellow and turquoise clusters corresponded to unaffected tissue. Blood and matrix were represented by the purple and blue clusters, respectively. (**B**) Based on the segmentation results different ROIs were selected for proteomic analysis. The colored regions (approximately 0.5 mm^2^) were ablated using LMD. Biol rep = biological replicate.

Spatial segmentation analysis was used to differentiate regions in the cardiac tissue based on spectral differences obtained by MALDI-MSI. As shown in Figure 3A, seven clusters were found, and each color depicts a particular cluster, of which five corresponded to the tissue, and two related to the blood and matrix (spectra can be found in Figure S6). In more detail, tissue-specific clusters, as confirmed with histological annotations (Figure 2D), showed that the unaffected tissue could be separated into red and yellow clusters, depending on the amount of interstitial stromal tissue (Figure S4D,E). Green and orange clusters corresponded to the infarct core and border tissue, respectively, with different levels of necrotic tissue damage. Within the core region hemorrhagic necrosis and inflammatory cell infiltration were observed (Figure S4G,H), whereas the border area mainly contained necrotic tissue with edema (Figure S4F). The purple cluster was defined as blood based on the presence of *m*/*z* 15198, which can be assigned to hemoglobin α-chain [25,26] and the matrix (blue) cluster was outside the tissue.

### 2.3. Image-Guided Proteomics Revealed Up- and Downregulation of Specific Proteins in the Infarct Area

**Figure 4:**
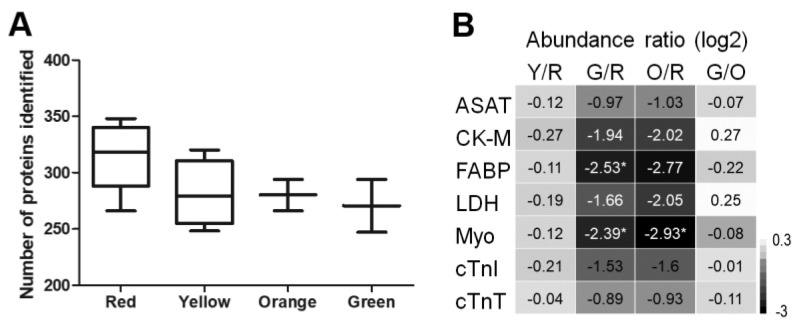
Identified proteins in the different clusters. (**A**) The number of proteins identified in the cluster replicates, and (**B**) heatmap showing the abundance ratio (log2) of classically known cardiac biomarkers. * indicates adjusted *p*-value ≤ 0.05. R = Red; Unaffected tissue, Y = Yellow; Unaffected interstitial stromal tissue, O = Orange; Infarct border, G = Green; Infarct core. Where Y/R is the ratio of Yellow versus Red. ASAT = Aspartate aminotransferase, CK-M = creatine kinase-M type, FABP = fatty acid-binding protein, LDH = lactate dehydrogenase, Myo = myoglobin, cTnI = cardiac troponin I, cTnT = cardiac troponin T.

Direct identification of proteins using MALDI-TOF-MSI is challenging due to the lack of chromatographic separation and protein dynamic range. To overcome this challenge, we used MALDI-MSI to screen the spatial protein content and later performed protein identification following a spatial-omics approach including LC-MS/MS, on the same tissue sections previously used for protein MALDI-MSI. Guided by the segmentation data, regions of 0.5 mm^2^ were ablated with laser microdissection from ITO slides: five red, four yellow, two orange and two green samples (colored areas in Figure 3B). Proteomic analysis identified a total of 465 proteins directly from the ITO slides; these proteins were used for subsequent comparative analysis (protein abundances can be found in Table S2). The number of identified proteins showed no significant differences between clusters, (ANOVA *p*-value = 0.34, Figure 4A).

First, abundance changes in (previously) used cardiac biomarkers that clinically serve for diagnosis and monitoring of MI such as cardiac troponins (cTnI and cTnT) were evaluated. The abundance ratios of these proteins are shown in the heatmap in Figure 4B and Figure S7. A significant downregulation was observed for myoglobin (MYO), creatine kinase-M type (CK-M) and fatty acid binding protein (FABP) in the infarct region.

**Figure 5:**
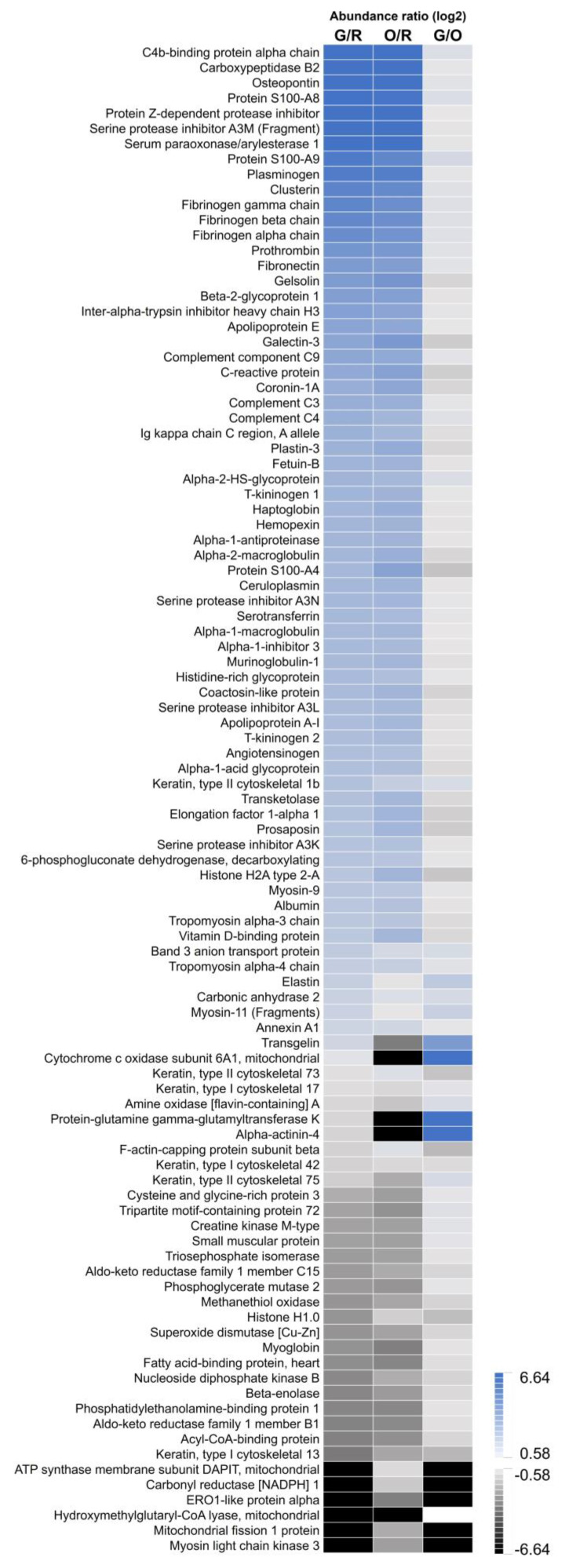
Heatmap showing the abundance ratios for the differential proteins (n = 99). In blue the upregulated proteins (log2 ratio ≥ 0.58), the downregulated proteins (log2 ratio ≤ −0.58) in black. R = Red; Unaffected tissue, O = Orange; Infarct border, G = Green; Infarct core. G/R is the ratio of Green versus Red, O/R is the ratio of Orange versus Red, G/O is the ratio of Green versus Orange.

From the identified proteins, 99 were found to be differentially abundant (log2 ratio ≤ −0.58 or ≥ 0.58 with an adj. *p*-value ≤ 0.05) in one of the comparisons as shown in Figure 5 (Table S3, Figures S8 and S9). It should be noted that 16 proteins were not detected in all regions, resulting in a minimal (black; −6.64) or maximal (dark blue; 6.64) abundance ratio in the heatmap of Figure 5.

A comparison of the unaffected tissue (red) with the infarct core (green) showed a lower abundance of 17 proteins, MYO and FABP amongst others cardiac biomarkers. Likewise, 56 proteins were upregulated, such as c-reactive protein an important diagnostic marker for inflammation, and indicators for cell damage: clusterin and protein S100. The heatmap in Figure 5 illustrates the comparison of the infarct regions (green or orange) with the unaffected tissue (red or yellow), resulting in similar protein patterns. Within the unaffected tissue, different abundances were found for 49 proteins between the yellow and red clusters. The significantly upregulated proteins in the interstitial stroma (e.g., capillaries and fibroblasts) were related to coagulation and a downregulation in cytoskeletal modulation.

More specifically for the ischemic region, a significant difference was found for 13 proteins between the border (orange) and core (green) infarct. The core region showed a higher abundance of structural proteins, such as elastin and transgelin, and a lower abundance of mitochondrial fission 1 protein and keratin, type I cytoskeletal 13 proteins.

Furthermore, pathway analysis was performed for the significantly altered proteins using the Reactome database through EnrichR (Table S4). The enriched pathways in the infarct core region compared to the unaffected tissue (green vs. red, Table 1) showed that the top 10 pathways are related to coagulation, inflammatory responses and integrin signaling.

Also, the comparisons within the infarct tissue (core-green vs. border-orange, Table 2) showed pathways involved in RHO GTPase activation and prostaglandin synthesis demonstrating ongoing changes in the architecture and inflammation. Only the synthesis of prostaglandin (PG) and thromboxane (TX) pathways were downregulated with a significant *p*-value.

Finally, a comparison was made between the MALDI-MSI dataset LC-MS/MS results and the literature. Several peaks were assigned to histone H4 (*m*/*z* 11309) [17,25], histone H2B (*m*/*z* 13775) [17], histone H2A (*m*/*z* 14008) [17], histone H2A Ac (*m*/*z* 14047) [17] and hemoglobin α (*m*/*z* 15189) [25,26].

## 3. Discussion

In this study we showed for the first time an intact protein-based spatial-omics approach on cardiac tissue sections from a single conductive ITO slide. In short, as a proof-of-concept, a cardiac I/R rat model was used for a MALDI-MSI-based unbiased evaluation of the spatial protein profiles, compared to classical pathological evaluation. The subsequent in-depth label-free proteomics analysis of selected regions provided insight in protein alterations and corresponding processes that play a role in the acute phase after I/R.

Previous studies of early phase ischemia in mouse [9] and pig [7] models showed the activation of immunological and inflammatory processes, alteration in the expression of structural proteins and the formation of edema in the infarct region. In addition, they observed alterations of contractile and mitochondrial proteins in the remote tissue. In our acute I/R model, we confirmed the increase in proteins related to coagulation and inflammatory processes in the infarct tissue (Table S4). Furthermore, changes in structural proteins were observed, and our histological annotations later confirmed the formation of edema. However, our unsupervised analysis did not show a distinct signature for the remote tissue in the I/R hearts compared to the control.

In addition, our MALDI-MSI data showed a differentiation within the infarct tissue of the core and border regions, where differences in extent of necrosis were verified by H&E staining (Figure S4). However, we were not able to differentiate between the different forms of cell death [27]. Specific immunohistochemistry and higher MSI spatial resolution would be relevant and should be considered in a follow-up study. The border or peri-infarct region has been described to play an important role in healing, but also in arrythmia development [11,12]. Since the early phase after I/R is mostly characterized as infarct expansion, most studies investigate this border area in a later stage when remodeling has started. In a pig model one month after I/R, the impairment of the energy metabolism, mitochondrial dysfunction and the activation of angiogenesis was found in the peri-infarct region indicating a downregulation of proteins in the electron transport chain in line with a decreased energy metabolism [12]. In comparison, our early I/R model showed a non-significant decrease in the abundance of mitochondrial membrane proteins. We hypothesize that our model studies an earlier phase in the remodeling process; the addition of multiple timepoints would provide temporal information on the effects of I/R injury. Although we used a small sample size (n = 3) for this proof-of-concept study, we were able to demonstrate the use of our spatial-omics workflow [22] on rat cardiac I/R tissue where we could verify previously published pathway alterations.

Even though our data identified a significant alteration of only 13 proteins between the core and border regions, it did demonstrate important differences and intra-infarct heterogeneity. For example, elastin is a protein involved in ECM organization, which is known to be changed in response to ischemia as the necrotic cells are replaced by ECM and later a fibrotic scar [28,29]. In addition, myosin-11 and transgelin were altered in the infarct; these proteins are known to be part of the vascular smooth muscle cell cytoskeleton. Interestingly, transgelin was upregulated in the infarct core compared to its border, while the border showed a significant downregulation compared with the unaffected tissue. Transgelin was previously indicated as a potential therapeutic target for MI, as it plays a role in NF-κB activity and vascular inflammation [30,31]. Future research into the different regions within the infarct will provide further information on the role of these proteins and the affected pathways. The use of a spatial-omics approach is beneficial to achieve this, as the boundaries of these regions might not be easily distinguishable when only based on their morphology.

As previously mentioned, histological annotation verified the segmentation results obtained by MALDI-MSI, showing differences in morphology of the unaffected tissue and in the extent of necrosis in the infarct tissue. Within the unaffected tissue, the different amounts of interstitial stromal tissue with fibroblasts and microvasculature most likely caused the distinction between the red and yellow clusters (Figure 4). This could be due to sectioning at slightly different levels in the heart, where the orientation of the cardiomyocytes differs, and more interstitial stromal tissue might be present. The higher abundance of proteins related to inflammation and coagulation might be the result of microvasculature activation in the interstitial stromal compartment in comparison to the unaffected tissue. Another explanation could be the so-called acute surgical trauma, previously described as an increased inflammation response in control animals [32].

The success of I/R surgery was confirmed with the significant increase in the biomarkers cTnT and cTnI in the blood circulation. However, our proteomics data did not reveal a significant decrease in these structural cardiac proteins in the infarct region compared to the unaffected tissue. Previous studies by gel electrophoresis are in line with our results where changes in cTnT after MI in dogs and human were not evident [33]. Another study found significant changes in cTnT in the cytosolic fraction after permanent ligation but not in an I/R model [9].

Furthermore, the inclusion of three biological replicates illustrated the variation in morphology of the hearts, mainly in the size of the infarct area. These differences are hypothesized to be the individual responses to the I/R surgery, where the exact placement of the suture and time of occlusion are known for their direct influence on the severity of the I/R injury. Although differences in the morphology and molecular profiles among hearts were observed, our MALDI-MSI analysis identified regions in the tissue that behaved similarly (in the same segmentation cluster).

Unfortunately, we were only able to provide a few identifications of some *m*/*z* values observed in our MALDI-MSI data due to previously described limitations of on-tissue protein analysis with MALDI-MSI. We obtained those by comparing protein identifications from LC-MS/MS with our protein MALDI-MSI data and the literature. Although there are differences between LC-MS/MS and MALDI-MSI; bottom-up versus top-down, as well as differences in ionization, analyte extraction and additional chromatographic separation, similar results should be expected for the most abundant proteins (for example hemoglobin). Additional experiments, such as top-down proteomics or MALDI-FTICR-MSI (as described by Piga et al. [34] and Dillilo et al. [35]), might be used in future studies to improve on-tissue protein identifications. Experiments using the same analyzers for both MALDI and LC-MS/MS analysis or MALDI-based proteomics would enable the correlation between the datasets regarding the protein identification. Nevertheless, the method we propose combines rapid MALDI-MSI and LC-MS/MS.

Regarding the LMD approach, some parts of the workflow need further consideration for future improvements. Firstly, a similar amount of tissue was collected for all ROIs, which was an area of a size that was based on previous results of healthy cardiac tissue [22]. Increasing the amount of tissue or the incorporation of an additional dimension in peptide separation will improve the number of proteins identified. Furthermore, no protein quantitation (for example using a Bradford assay) was performed prior to LC-MS/MS due to the limited amount of samples. Nevertheless, between the ablated areas a comparable number of peptides was detected, and a similar number of proteins was identified (ANOVA non-significant). We previously showed that a spatial-omics approach works efficiently on conductive ITO slides and although the number of proteins identified after MALDI-MSI decreased compared to before MALDI-MSI, the cellular component analysis showed that the proteins were preserved [22]. However, while the use of a consecutive section on PEN membrane slides provides more proteins identified, the use of a consecutive section hampers proper co-registration in some cases, as the morphology might be different between slides. Co-registration is crucial for proper tissue selection, especially with small ROIs, such as in our study. Here, minor spatial inaccuracy might result in limitations when selecting different proximal areas. Additionally, further improvements of the co-registration might facilitate the selection of specific cells and cell populations, thereby facilitating the evaluation of cell-specific contributions to the pathological changes after I/R. This approach was previously used in a gene expression study using laser microdissection pressure catapulting, showing an upregulation of p21 in cardiac fibroblasts and cardiomyocytes in the peri-infarct zone. Finally, adjustments to the proteomics protocol might improve the evaluation of certain ECM proteins. In this case, the use or addition of another enzyme, such as collagenase, might result in more ECM proteins being identified as well as the detection of post-translational modifications [36,37,38]. As edema formation and extra cellular matrix remodeling play an important role after an ischemic event, it is expected that this might provide more insight into the involved proteins.

In the present study, an acute I/R model was used, providing information on the processes initiated early after I/R. Future research should be considered for the translation from a (small) animal model to a human model: both on the level of the proteome and pathological responses between species, as well as the inclusion of comorbidities. Other experimental work including immunohistochemical staining and/or MALDI-FTICR-MSI would be of added value, as on-tissue identification of proteins remains challenging with MALDI-MSI.

## 4. Materials and Methods

### 4.1. Chemicals and Solvents

All solvents (ULC grade) were purchased from Biosolve (Valkenswaard, The Netherlands) unless stated otherwise. Ammonium bicarbonate (ABC), 2,6-dihydroxyacethophenone (DHA), dithiothreitol (DTT), Eosin-Y (Avantor), formic acid (FA, ULC grade), Gill’s hematoxylin, iodoacetamide (IAM), trifluoroacetic acid (TFA, ULC grade), and xylene were purchased from Sigma-Aldrich (Zwijndrecht, The Netherlands). RapiGestTM SF was purchased from Waters (Milford, CT, USA). Trypsin (Modified porcine, Sequencing Grade) was purchased from Promega (Leiden, The Netherlands). In addition, 0.2 mL centrifuge tubes were purchased from Leica Microsystems (Wetzlar, Germany). Indium tin oxide (ITO) glass slides were obtained from Delta Technologies (Loveland, CO, USA).

### 4.2. Tissue and Blood Samples

All animal experiments were performed according to the Guide for the Care and Use of Laboratory Animals (U.S. National Institutes of Health publication 85-23, revised 1996) and was approved by the local ethical committee (CENOMEXA number 54; ref 0871.01). For this study, six 12-week-old male Wistar rats (Janvier Labs, Le Genest-Saint-Isle, France) were subjected to either sham surgery or ischemia/reperfusion (n = 3/group), as described before [39,40]. In short, the animals were anesthetized with an intraperitoneal administration of ketamine/xylazine (150 and 5 mg/kg, respectively). Ischemia was provoked by the temporary occlusion of the proximal left coronary artery (40 min), followed by reperfusion for 24 h (visually verified before closing the chest). After 24 h the animals were sacrificed and the hearts were excised, transversally cut and washed in ice-cold PBS buffer to remove the blood. The hearts were immediately frozen in liquid nitrogen and kept at −80 °C until analysis. The tissue was sectioned at 10 µm thickness using a cryotome (Leica Microsystems, Wetzlar, Germany) at −20 °C, sections were thaw-mounted on ITO glass slides and stored at −80 °C until further analysis. The blood was collected from the abdominal aorta and collected in lithium heparin or EDTA plasma tubes, centrifuged, and stored at −80 °C until further analysis.

### 4.3. Clinical Chemistry Measurements

High-sensitivity cardiac troponin T and I concentrations (hs-cTnT and hs-cTnI, respectively) were measured in the obtained plasma samples with the hs-cTnT STAT immunoassay on the Cobas e602 (Roche Diagnostics, Mannheim, Germany) and the hs-cTnI STAT immunoassay on the Alinity i (Abbott Diagnostics, Wiesbaden, Germany). The unpaired t-test was used to compare the concentrations between the sham and I/R samples, a *p*-value ≤ 0.05 was considered statistically significant.

### 4.4. Intact Protein MALDI Mass Spectrometry Imaging

Tissues were washed for 30 s in 70% ethanol, 30 s in 100% ethanol, 2 min in Carnoy’s solution (being 60% ethanol, 30% chloroform and 10% acetic acid), followed by 30 s in 100% ethanol, demineralized water and 100% ethanol. Afterwards, they were dried in a desiccator. Next, 9 layers of 15 mg/mL DHA in 80% acetonitrile, 0.4% TFA and 0.4% acetic acid were applied using the SunCollect sprayer (SunChrom GmbH, Friedrichsdorf, Germany). For co-registration purposes, fiducial markers were placed next to the tissue using water-based Tipp-Ex (BIC, Paris, France). The tissue was analyzed with a RapiFleX Tissuetyper (Bruker Daltonics GmbH, Bremen, Germany) in positive-ion linear mode, summing 1000 laser shots per position with a laser frequency of 5000 Hz and 80 µm pixel size. Data were acquired in the *m*/*z* range from 2000–20,000 and protein calibration standard I (Bruker Daltonics) was used for instrument calibration. Slides were stored at −80 °C until LMD.

### 4.5. MSI Data Analysis

All datasets were recalibrated using FlexAnalysis v3.4 (Bruker Daltonics) for optimal spectral comparison. This was performed in quadratic correction mode using *m*/*z* 5487, 8565, 11,307, 12,135 and 15,198 as calibrants with a 500 ppm peak assignment tolerance. After recalibration, the MSI data was analyzed using SCiLS lab MVS version 2021b (Bruker, Bremen, Germany) after total ion current (TIC) normalization. *m*/*z* images were generated with an interval width of 2 Da. The overall average spectrum was imported in mMass [41] to generate a peak list (50 precision baseline correction with 75 relative offset, moving average smoothing window of 5 *m*/*z* and 2 cycles, S/N threshold of 2.5, relative intensity threshold of 1.0% and picking height 75). Probabilistic latent semantic analysis (pLSA) [42] with 5 components was performed with random initialization using the overall peaklist on the individual spectra. Discriminative *m*/*z* values were evaluated using receiver operating characteristic (ROC) analysis [43] comparing the infarct and unaffected regions from I/R and sham hearts as found by the pLSA taking a random subset of 3000 spectra. The area under the curve (AUC) threshold ≥0.8 or <0.2, resulted in *m*/*z* values specific for the infarct or the unaffected regions, respectively. Segmentation was performed in SCiLS using bisecting k-means with the over-all peaklist and correlation distance, to obtain region of interest (ROI) information. The coordinates from the ROIs were exported for LMD using an in-house build MATLAB script [44].

### 4.6. Laser Capture Microdissection

From the selected ROIs, areas of 0.5 mm^2^ were dissected using the Leica LMD 7000 (Leica Microsystems, Wetzlar, Germany) using the previously established protocol [22], with the following laser settings: wavelength 349 nm, power 40, aperture 38, speed 5, specimen balance 0, line spacing 5, head current 60%, and pulse frequency 310Hz in “draw + scan” mode. Different methods for tissue collection were compared, see Figure S1. Based on these results, the dissected tissue was chosen to be collected without prior removal of the DHA matrix in 0.2 mL centrifuge tubes containing 20 µL of ethanol, the sample was dried in the SpeedVac and resuspended in 20 µL of 50 mM ABC buffer and stored at −20°C until further processing.

### 4.7. Sample Processing for Proteomics

The dissected material was further processed as previously described [22]. In short, 2.2 µL of 0.1% RapiGestTM was added to every sample to enhance enzymatic protein digestion and incubated for 10 min at room temperature (RT = 21 °C). The samples were reduced by adding 1.3 µL of DTT (200 mM in 50 mM ABC) at 56 °C for 40 min and 800 rpm in a Thermoshaker (Eppendorf, Hamburg, Germany) and alkylated by adding 1.4 µL of IAM (400 mM in 50 mM ABC) at RT for 30 min and 800 rpm. To quench the excess of IAM, 1.4 µL of DTT (200 mM in 50 mM ABC) was used at RT for 10 min at 800 rpm. Protein digestion was performed using a double trypsin step; the first overnight step at 37 °C and 800 rpm was conducted after adding 1 µL of trypsin (0.5 µg/µL), the second step was performed after adding 0.3 µL of trypsin (0.5 µg/µL) and 115 µL of ACN at 37 °C for 3 h at 800 rpm. Digestion was stopped by adding 6 µL of TFA (10%) at 37 °C for 45 min at 800 rpm. After centrifugation (150,000× *g*, 10 min at 4 °C, Thermo Scientific Heraeus Biofuge Stratos, Waltham, MA, USA), the supernatant was concentrated using a SpeedVac (Hetovac VR-1, Heto Lab Equipment, Denmark) to a final volume of 30 µL. The samples were stored at −20 °C until LC-MS/MS analysis.

### 4.8. LC-MS/MS Anasis

An aliquot of 10 µL of the sample was used for the LC-MS/MS analysis as previously described [22]. In short, an online installed C18 trapping column was used for desalting. This was performed on a Thermo Scientific (Dionex, Sunnyvale, CA, USA) Ultimate 3000 Rapid Separation UHPLC system. and a PepSep C18 analytical column (15 cm, ID 75 µm, 1.9 µm Reprosil, 120 Å) was used to separate the peptides with a flowrate of 300 nL/min, solvent A (100% H_2_O, 0.1% FA) and solvent B (80% ACN, 0.1% FA) and a 110 min linear gradient from 5% to 32% ACN with 0.1% FA as follows: 0–3 min 0% B; 3 min 5% B; 103 min 27.5% B; 113 min 40% B; 114–120 min 95% B; 121–140 min 3% B. The UHPLC system was coupled to a Q ExactiveTM HF mass spectrometer (Thermo Scientific) with Nanospray Flex source. Mass spectra were acquired in positive ionization mode, with a full MS scan between 250–1250 *m*/*z* at resolution of 120,000 followed by MS/MS scans of the top 15 most intense ions at a resolution of 15,000 in DDA mode.

### 4.9. Proteomics Data Analysis

Protein identification was performed using Proteome Discoverer 2.2 (Thermo Scientific). The search engine Sequest was used with the SwissProt Rattus norvegicus (SwissProt TaxID = 10116) database, August 2020. The database search was performed using trypsin as an enzyme and a maximum of 2 missed cleavages. The minimal peptide length was set to 6 amino acids, with a mass tolerance for precursors of 10 ppm and for fragments of 0.02 Da. Methionine oxidation and protein N-terminus acetylation were set as dynamic modifications and carbamidomethylation of cysteine residues as a static modification. Proteins were identified based on the peptide spectrum match (PSM) score and protein interference was used to create protein groups with unambiguous and unique peptides. The false discovery rate was fixed at 1% and used as a measure for certainty of the identification; only proteins with a high protein confidence (99% accuracy) were used for further analysis. The number of identified proteins for the segmentation clusters was presented as median ± inter-quartile range (IQR). The variation regarding the numbers of proteins identified was analyzed using an ANOVA test.

The Proteome Discoverer software was used for further analysis of the identified proteins. It was used to determine protein abundances and calculate the abundance ratios between clusters, such as ‘green/red’ or ‘yellow/red’. Next, the ANOVA test was performed to determine if the selected ratios show a statistically significant change. Only protein abundance ratios with a fold-change higher than 1.5 or lower than 0.67 (log2 ≥ 0.58 or ≤ −0.58, respectively) and adjusted *p*-value ≤ 0.05 were considered for further analysis. For the pathway analysis, protein accession numbers were converted to gene names using UniProt ID mapping. The significantly altered proteins were included in the pathway analysis using EnrichR [45,46] with Reactome’s cell signaling database. The up- or downregulated pathways (with adjusted *p*-value < 0.05) were ranked by the combined score.

### 4.10. Haematoxylin and Eosin (H&E) Staining

After LMD a standard H&E staining protocol was performed [22], which included a 3 min nuclei stain with 0.1% Gill’s hematoxylin and a 30 s cytoplasm stain with 0.2% eosin. The H&E-stained samples were mounted with a cover slip and a digital optical image was obtained using the Aperio CS2 slide scanner (Leica Microsystems, Wetzlar, Germany). Annotation of various areas was conducted on consecutive sections and performed by a pathologist using QuPath v0.2.3 [47].

## 5. Conclusions

With this proof-of-concept work we showed the usefulness of a protein-guided spatial-omics approach using conductive ITO slides for cardiovascular research. We characterized significant local changes in the protein content of the infarct core and border regions compared to unaffected tissue in rat hearts after I/R. Future experiments should include multiple timepoints and a permanent cardiac ischemia model. Future research should place efforts into protein identification after protein MALDI-MSI, using targeted top-down approaches. Another way forward would be to ensure proper correlation between the datasets, using peptide MALDI-MSI or MALDI-based proteomics for swift comparison of protein identifications as well as the use of high mass resolution MALDI-FTICR-MSI. Overall, our results demonstrate that our approach can also be applied to other cardiac models, such as a permanent ligation or heart failure model, providing valuable insights into the protein-driven pathophysiological processes after cardiac injury.

## Figures and Tables

**Table 1:** Top 10 upregulated pathways for the infarct core compared to unaffected tissue (green vs. red), with an adjusted *p*-value < 0.05 and ranked by the combined score ^1^.

Upregulated Pathways	Overlap	Combined Score
Platelet degranulation	16/105	5135.92
Response to elevated platelet cytosolic Ca^2+^	16/110	4791.00
GRB2:SOS provides linkage to MAPK signaling for Integrins	4/15	2403.01
p130Cas linkage to MAPK signaling for integrins	4/15	2403.01
Platelet activation, signaling and aggregation	17/253	1679.42
Common pathway of fibrin clot formation	4/22	1320.54
Terminal pathway of complement	2/8	1089.78
Platelet aggregation (Plug Formation)	5/37	1069.55
Formation of fibrin clot (Clotting Cascade)	5/39	990.15
Integrin alphaIIb beta3 signaling	4/27	973.26

^1^ Reactome pathway analysis was conducted including the significantly altered proteins from the infarct core vs unaffected tissue (green vs. red). The overlap indicates the number of proteins identified compared to the number of proteins in the pathway.

**Table 2:** Upregulated pathways for the infarct core compared to infarct border (green vs. orange), with an adjusted *p*-value <0.05 and ranked by the combined score ^1^.

Upregulated Pathways	Overlap	Combined Score
RHO GTPases activate CIT	1/16	1422.78
RHO GTPases Activate ROCKs	1/17	1318.68
RHO GTPases activate PAKs	1/21	1012.63
Nephrin interactions	1/22	955.53
Sema4D induced cell migration and growth-cone collapse	1/24	857.29
Sema4D in semaphorin signaling	1/27	740.22
Smooth Muscle Contraction	1/33	576.31
EPHA-mediated growth cone collapse	1/34	555.22
Elastic fibre formation	1/41	439.30

^1^ Reactome pathway analysis was conducted including the significantly altered proteins from the infarct core vs. unaffected tissue (green vs. red). Overlap indicates the number of proteins detected compared to the number of proteins in the pathway.

## Data Availability

The mass spectrometry proteomics dataset can be found in the ProteomeXchange Consortium via the PRIDE 46 partner repository with the dataset identifier PXD026115.

## Supplementary Materials

The supporting information can be downloaded at: https://www.mdpi.com/article/10.3390/ijms232213847/s1.
