# Supplementary material for: Protein Alterations in Cardiac Ischemia/Reperfusion Revealed by Spatial-Omics
Source: Int J Mol Sci. 2022 Nov 10;23(22):13847. doi: 10.3390/ijms232213847 (PMC9692276; doi:10.3390/ijms232213847)
Supplement: Supplementary file 1 [file ijms-23-13847-s001.zip › ijms-1959671-supplementary.pdf]

Supporting information

Protein alterations in cardiac ischemia/reperfusion revealed by spatial-omics

Stephanie T.P. Mezger [1,2,3], Alma M.A. Mingels [2,3], Matthieu Soulié [4], Carine J. Peutz-Kootstra [5], Otto Bekers [2,3], Paul Mulder [4], Ron M.A. Heeren [1] and Berta Cillero-Pastor [1,6,*]

1 Maastricht MultiModal Molecular Imaging (M4i) Institute, Division of Imaging Mass Spectrometry, Maastricht University, Universiteitssingel 50, 6229 ER Maastricht, The Netherlands
2 Central Diagnostic Laboratory, Maastricht University Medical Center, P.O. Box 5800, 6202 AZ Maastricht, The Netherlands
3 CARIM School for Cardiovascular diseases, Maastricht University, Universiteitssingel 50, 6229 ER Maastricht, The Netherlands
4 Normandie Université, UNIROUEN, Inserm U1096, Endothelium, Valvulopathy & Heart Failure, 76183, Rouen, France
5 Pathologie Friesland, 8901, EN, Leeuwarden, The Netherlands
6 MERLN Institute for Technology-inspired Regenerative Medicine, cBITE – Cell Biology-Inspired Tissue Engineering department, Maastricht University, Universiteitssingel 40, 6229 ER Maastricht, The Netherlands
* Correspondence: b.cilleropastor@maastrichtuniversity.nl

Figures

Figure S1: Comparison of tissue collection methods after protein MALDI-MSI
Figure S2: Schematic representation of the workflow
Figure S3: Probabilistic latent semantic analysis (pLSA) separating the data in 5 components
Figure S4: Histological annotation of the I/R hearts
Figure S5: ROC analysis provides m/z values that separate infarct from unaffected tissue.
Figure S6: Average spectra corresponding to all clusters from the segmentation analysis
Figure S7: Heatmap of classically known cardiac biomarkers for infarct regions vs unaffected interstitial stromal tissue
Figure S8: Heatmap of grouped abundances for the differentially abundant proteins (n=99)
Figure S9: Heatmap of differentially abundant proteins (n=99) for infarct regions vs unaffected interstitial stromal tissue

Tables

Table S1: ROC analysis revealed distinctive m/z values for the different regions within the I/R and sham hearts
Table S2: Protein abundances for all sample (separate excel)
Table S3: Significant altered proteins (n=99) found in the proteomics data
Table S4: Pathways analyses including the significantly up- or downregulated proteins (separate excel)

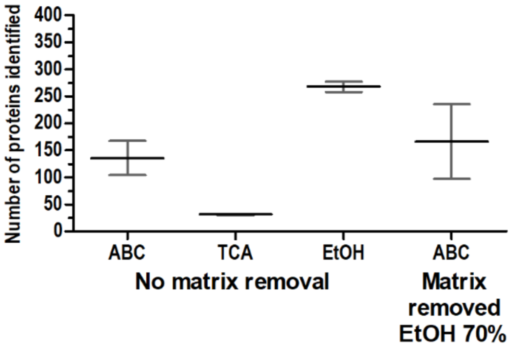

Supporting Figure S1. Comparison of collection methods and number of identified proteins after MALDI-MSI.

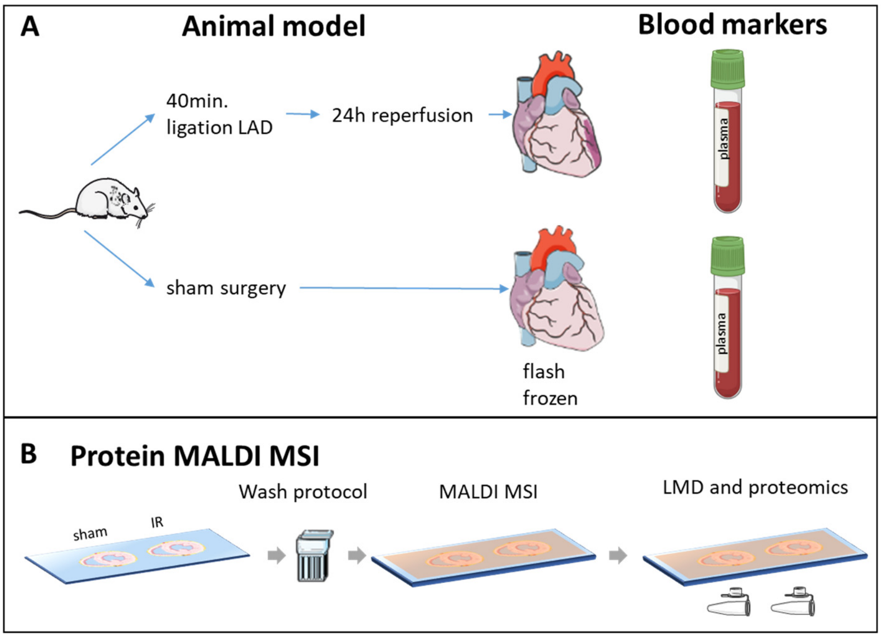

Supporting Figure S2. Schematic representation of the workflow. A) Wistar rats underwent sham or I/R surgery (n=3/group). Ischemia was induced by 40 min ligation of the left anterior descending (LAD) artery followed by 24 hours of reperfusion, after which both the heart and blood were collected for further analysis; B) The cardiac tissue was sectioned at 10 µm thickness, deposited on ITO slides, washed and covered with DHA matrix for protein MALDI-MSI followed by laser capture microdissection (LMD) and proteomics.

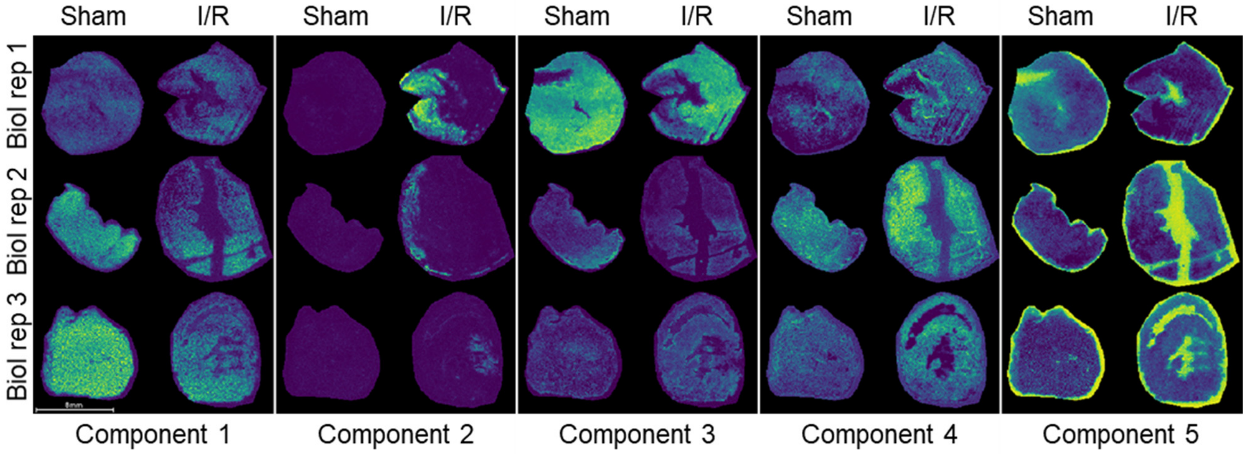

Supporting Figure S3. Probabilistic latent semantic analysis (pLSA) (5 components), revealed the separation of the tissue in unaffected myocardial tissue (components 1 and 3), infarct (component 2), tissue containing interstitial stroma (component 4) and matrix (component 5).

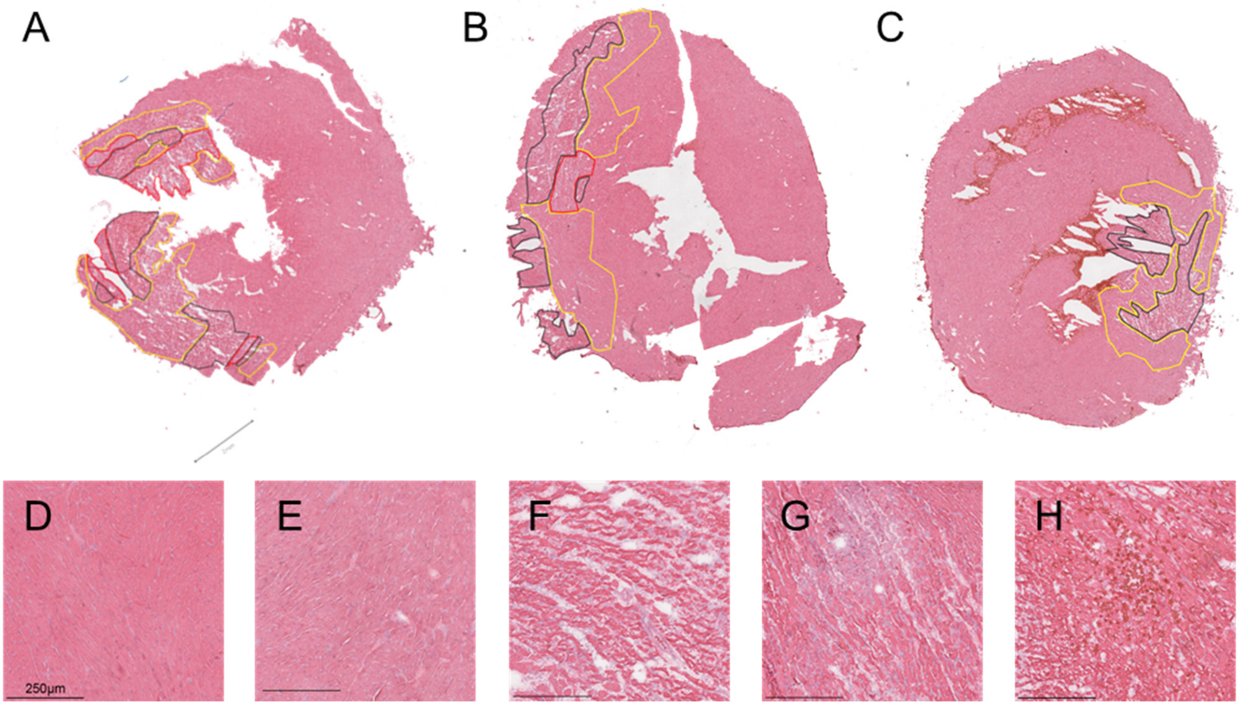

Supporting Figure S4. Histological annotation of the I/R hearts (consecutive sections, A-C) identified (D) unaffected myocardial tissue, (E) unaffected interstitial stromal tissue, (F) necrosis with edema (yellow in A-C), (G) necrosis with cell infiltration (red in A-B) and (H) Hemorrhagic necrosis (black in A-C). The scale bar in A-C indicates 2 mm and in D-H 250 µm.

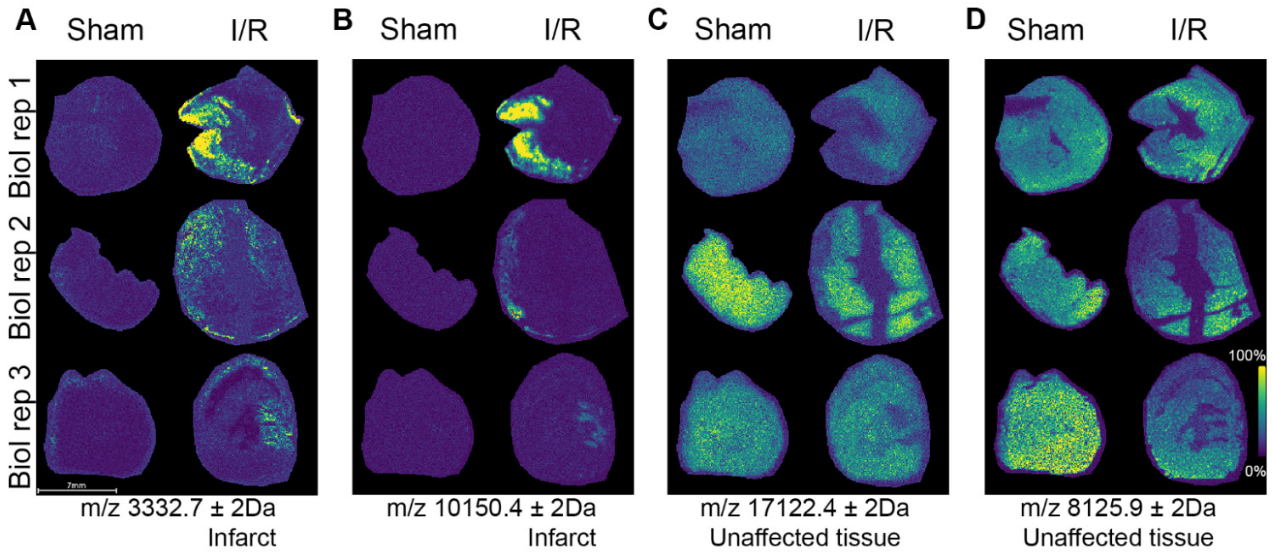

Supporting Figure S5. ROC analysis illustrated 55 m/z values that discriminate between the infarct tissue and the unaffected tissue from the I/R and sham hearts. Representative examples are A) m/z 3332.7 (AUC 0.939), B) m/z 10150.4 (AUC 0.935), C) m/z 17122.4 (AUC 0.097) and D) m/z 8125.9 (AUC 0.054). Biol rep = biological replicate

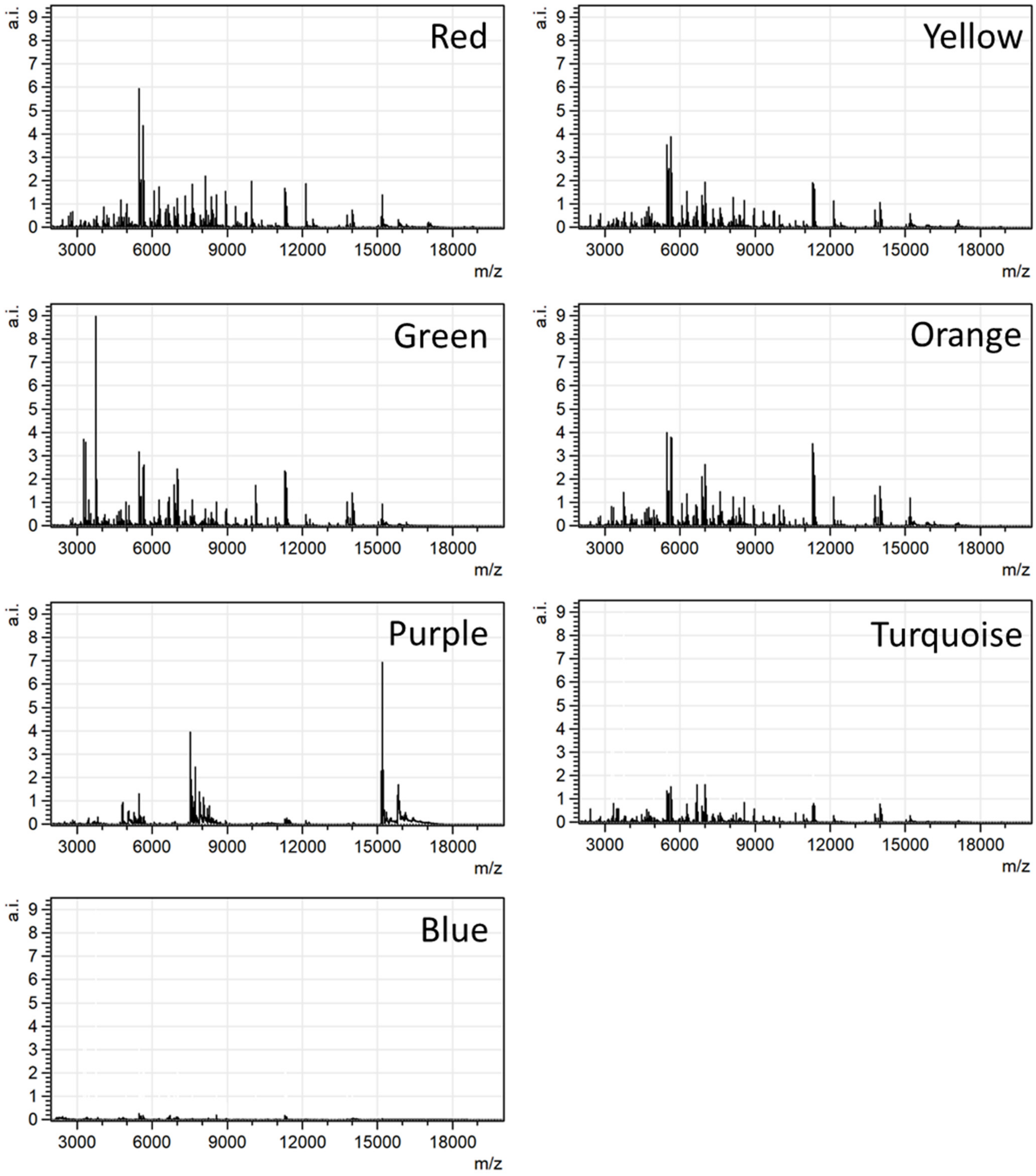

Supporting Figure S6. Average spectra corresponding to clusters obtained from the segmentation analysis (Figure 3). The unaffected tissue is represented by red, yellow and turquoise clusters. The infarct tissue by the green and orange clusters, purple represents the blood and blue the matrix.

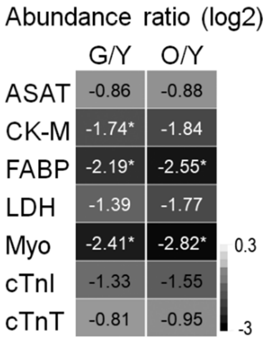

Supporting Figure S7. Heatmap showing the abundance ratio (log2) for classically known cardiac biomarkers for the infarct regions (G = Green; infarct core, O = Orange; infarct border) vs the unaffected interstitial stromal tissue (Y = Yellow).

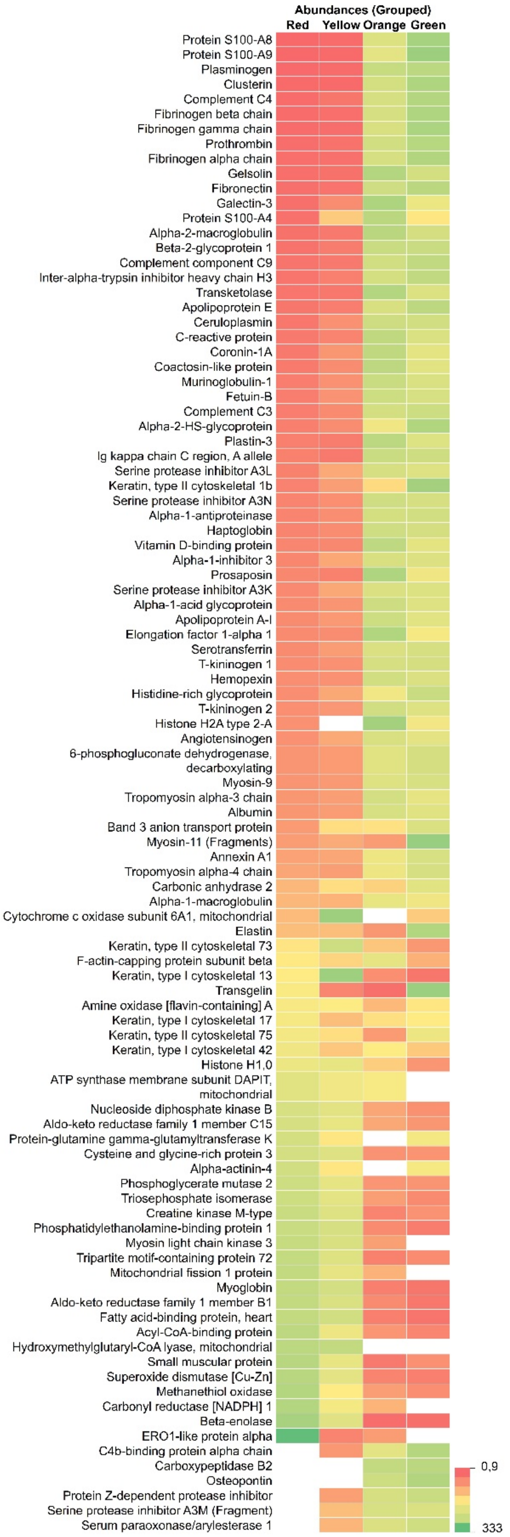

Supporting Figure S8. Heatmap showing the grouped abundances for all differentially abundant proteins (n=99) for the unaffected regions (Y = Yellow, unaffected stromal tissue, R = Red, unaffected tissue) and infarct regions (G = Green; infarct core, O = Orange; infarct border).

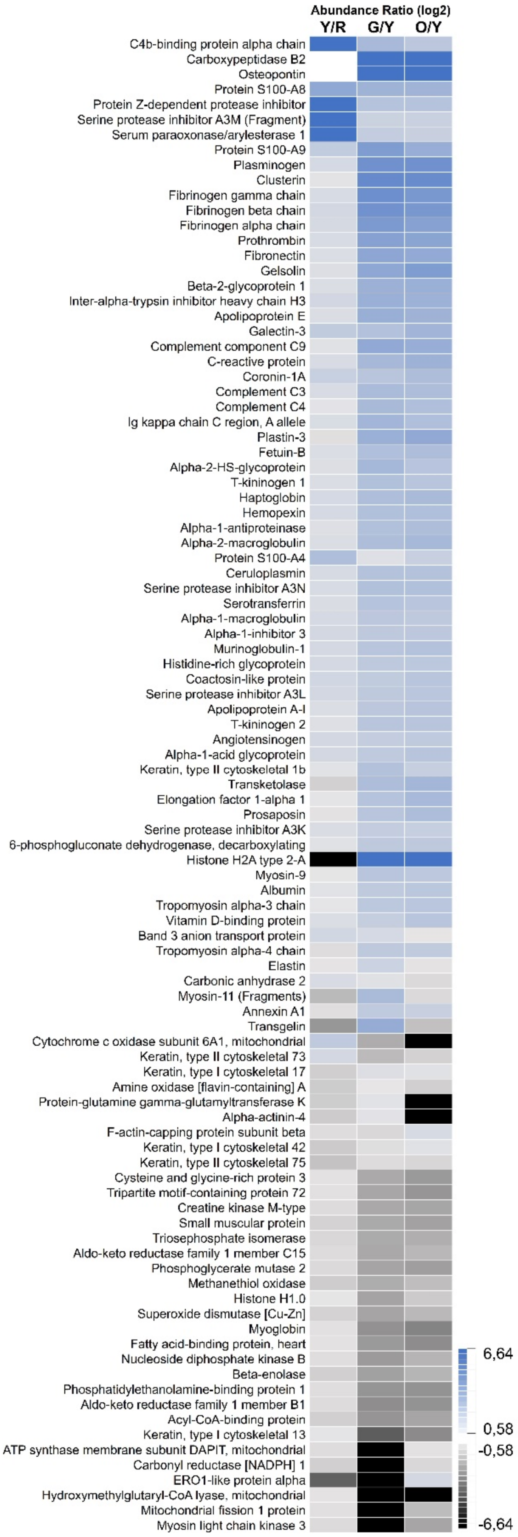

Supporting Figure S9. Heatmap showing the abundance ratio (log2) for all differentially abundant proteins (n=99) for the unaffected regions (Y = Yellow versus R = Red) and infarct regions (G = Green; infarct core, O = Orange; infarct border) vs the unaffected stromal tissue (Y = Yellow).

Supporting Table S1. ROC analysis, performed based on the pLSA, revealed distinctive m/z values for the different regions within the I/R and sham hearts. A comparison is made between the infarct (I/R) region and the unaffected tissue in the I/R (remote) and/or sham hearts. The AUC value was determined for the m/z values from the peaklist.

m/z	I/R vs Remote	I/R vs Sham	Remote vs Sham	m/z	I/R vs Remote	I/R vs Sham	Remote vs Sham
2021.3	0.4096	0.5400	0.6392	4964.9	0.4240	0.7098	0.7715
2220.8	0.4882	0.6215	0.6402	4990.2	0.1558	0.3154	0.5670
2360.8	0.3361	0.6100	0.7535	5074.5	0.7502	0.7372	0.4564
2380.8	0.4406	0.5402	0.5802	5487.6	0.3132	0.2447	0.4454
2393.5	0.4197	0.5607	0.6324	5504.5	0.2857	0.2919	0.4791
2419.8	0.1271	0.3553	0.7727	5520.0	0.1711	0.2480	0.5447
2657.6	0.4138	0.2942	0.3365	5535.7	0.1402	0.2766	0.6136
2673.5	0.2535	0.3544	0.6459	5551.6	0.1805	0.3376	0.6643
2741.7	0.2982	0.3105	0.4316	5637.7	0.1889	0.1650	0.4423
2765.3	0.3097	0.3744	0.5492	5655.3	0.6346	0.5214	0.3941
2816.9	0.2006	0.2831	0.4266	5675.5	0.6872	0.5215	0.3196
2833.4	0.3853	0.4152	0.5082	5906.1	0.3729	0.4269	0.5718
3111.1	0.4332	0.4926	0.5412	6068.2	0.1789	0.1369	0.3363
3137.8	0.2870	0.3635	0.5508	6221.5	0.2754	0.2281	0.4827
3162.4	0.4547	0.5765	0.6071	6238.9	0.3891	0.4083	0.5295
3178.1	0.3806	0.5780	0.7090	6278.3	0.2382	0.1672	0.5176
3266.9	0.9262	0.9241	0.5336	6295.3	0.1913	0.1413	0.4468
3332.7	0.8701	0.9390	0.7871	6327.9	0.4413	0.5688	0.6452
3456.6	0.3119	0.3356	0.4881	6541.3	0.3953	0.1576	0.2684
3465.9	0.8288	0.8727	0.5644	6650.0	0.3881	0.5921	0.6750
3479.3	0.3175	0.3996	0.6077	6666.2	0.4104	0.7199	0.8543
3508.7	0.4460	0.7149	0.7340	6683.5	0.3014	0.4128	0.5888
3525.3	0.3799	0.5755	0.6788	6699.3	0.3686	0.4459	0.5706
3665.8	0.4046	0.4448	0.5155	6714.2	0.3457	0.4261	0.5797
3761.9	0.8981	0.8915	0.4858	6894.6	0.7194	0.7196	0.5542
3785.3	0.6188	0.4594	0.3562	6916.6	0.3150	0.2389	0.3985
3832.7	0.6377	0.6431	0.4974	6951.0	0.4884	0.6767	0.7227
4044.7	0.4484	0.3179	0.3440	6964.3	0.3465	0.2947	0.4335
4062.4	0.2645	0.2001	0.2989	7005.2	0.4640	0.7716	0.8181
4120.1	0.6530	0.7720	0.6643	7020.3	0.6782	0.8768	0.8262
4184.1	0.3587	0.3272	0.4337	7082.7	0.6269	0.8174	0.7253
4199.3	0.3710	0.3582	0.4942	7333.3	0.1925	0.1788	0.4859
4227.6	0.5000	0.4685	0.4790	7365.1	0.1700	0.1993	0.5166
4283.3	0.2801	0.5139	0.7199	7514.9	0.4556	0.6622	0.7062
4464.7	0.2221	0.4411	0.5851	7535.5	0.5443	0.5854	0.5430
4472.7	0.1794	0.4574	0.7018	7578.9	0.4918	0.4574	0.4597
4485.1	0.1699	0.4113	0.7251	7601.5	0.4021	0.4058	0.5234
4597.0	0.6024	0.6017	0.5027	7653.3	0.3495	0.2216	0.4024
4669.4	0.3828	0.5248	0.6412	7671.9	0.4405	0.3583	0.4243
4766.9	0.2674	0.2544	0.5405	7929.4	0.3739	0.2687	0.3615
4783.1	0.1751	0.2709	0.6088	7958.9	0.3988	0.3569	0.4694
4815.0	0.3230	0.4071	0.5624	8042.3	0.2045	0.2417	0.5746
4831.4	0.3117	0.3959	0.5695	8058.7	0.1241	0.1718	0.5411
4861.2	0.3715	0.2918	0.3719	8125.9	0.1636	0.0536	0.2969
4876.5	0.2575	0.2482	0.4530	8142.5	0.2028	0.1366	0.3889

m/z	I/R vs Remote	I/R vs Sham	Remote vs Sham
8240.9	0.1371	0.3210	0.7392
8368.2	0.2943	0.2535	0.4613
8384.4	0.2272	0.2396	0.5088
8400.8	0.2159	0.3060	0.5942
8417.3	0.2438	0.3760	0.6192
8456.9	0.2927	0.1502	0.4113
8515.2	0.2000	0.1044	0.3717
8566.3	0.1555	0.2461	0.6996
8929.4	0.2040	0.3578	0.6469
8945.8	0.0757	0.3278	0.7732
8972.7	0.1332	0.2986	0.7722
9329.7	0.1574	0.0716	0.4061
9346.5	0.2382	0.1863	0.3895
9362.7	0.3258	0.2571	0.3892
9378.8	0.2419	0.2193	0.4393
9401.9	0.3776	0.2291	0.3535
9477.2	0.1945	0.1569	0.5086
9561.0	0.1939	0.1940	0.4756
9722.3	0.1828	0.0958	0.3339
9753.2	0.1262	0.0906	0.3709
9980.6	0.1515	0.2589	0.5998
10013.6	0.1876	0.3108	0.6087
10029.7	0.1944	0.2768	0.5831
10067.2	0.2082	0.1147	0.3423
10094.7	0.1869	0.1591	0.4338
10150.4	0.9347	0.9153	0.4373
10166.9	0.9320	0.9199	0.4625
10262.4	0.3854	0.2945	0.4017
10365.5	0.3169	0.1321	0.2739
10382.1	0.3133	0.2441	0.4142
10623.1	0.3803	0.7508	0.8730
10692.0	0.3037	0.2037	0.3613
10788.4	0.2316	0.2535	0.5264
10951.0	0.4019	0.7086	0.8011
11074.7	0.3685	0.5787	0.7354
11308.1	0.6229	0.5900	0.4582
11323.7	0.6209	0.7142	0.6269
11349.8	0.6682	0.6077	0.4293
11366.1	0.7392	0.7816	0.5763
11390.2	0.6916	0.6196	0.4204
11406.9	0.6872	0.6794	0.4994
11448.1	0.6145	0.5543	0.4322
11468.1	0.5454	0.5464	0.5051
12133.9	0.1663	0.1017	0.3492
12149.8	0.1293	0.0813	0.3037
12167.5	0.3445	0.2535	0.3424
12182.1	0.1902	0.1461	0.3209
12198.2	0.2491	0.1966	0.3984
12295.0	0.8025	0.7924	0.5046
12438.0	0.1752	0.1068	0.3714
12470.5	0.3475	0.2526	0.3732
12486.5	0.3553	0.2722	0.4122

m/z	I/R vs Remote	I/R vs Sham	Remote vs Sham
12565.6	0.3809	0.2575	0.3476
12582.5	0.4022	0.2869	0.3795
12598.8	0.4021	0.3075	0.3984
13422.2	0.5966	0.6525	0.5724
13470.2	0.4098	0.3102	0.3928
13776.2	0.5761	0.5672	0.5014
13791.3	0.7202	0.7559	0.6303
13900.3	0.5587	0.7643	0.7329
14007.1	0.4843	0.7564	0.8106
14030.8	0.6313	0.8640	0.8265
14046.9	0.7409	0.8923	0.8294
14070.8	0.7821	0.8369	0.5913
14087.0	0.6589	0.6899	0.5632
14109.0	0.6333	0.6706	0.5520
14422.4	0.5889	0.4772	0.3733
15027.0	0.4707	0.5872	0.6307
15154.5	0.4704	0.4339	0.4645
15198.8	0.4234	0.4229	0.5254
15245.3	0.3238	0.3906	0.5817
15313.7	0.5969	0.5228	0.4184
15356.9	0.6032	0.5289	0.4076
15821.0	0.4925	0.4087	0.4090
15852.9	0.3295	0.2256	0.3315
15905.5	0.4502	0.3310	0.3851
16155.9	0.5984	0.5959	0.4980
17028.1	0.1443	0.1007	0.3615
17122.4	0.0974	0.0873	0.4327
18805.0	0.3637	0.2230	0.3248

Supporting Table S2. Protein abundances for all samples.

Provided as excel document

Supporting Table S3. Significant altered proteins (n=99, figure 6) found in the proteomics data, the abundance ratio (log2) and the adjusted p-value are shown. R = Red; Unaffected tissue, Y = Yellow; Unaffected stromal tissue, O = Orange; Infarct border, G = Green; Infarct core. Where, for example, YR is the ratio of Yellow versus Red.

	Abundance Ratio (log2)						Abundance Ratio Adj. P-Value					
**Protein list**	**Y/R**	**G/R**	**G/Y**	**O/R**	**O/Y**	**G/O**	**Y/R**	**G/R**	**G/Y**	**O/R**	**O/Y**	**G/O**
6-phosphogluconate dehydrogenase, decarboxylating	0.3	2.06	1.69	1.95	1.7	0.16	0.372	0.030	0.040	0.200	0.212	0.922
Acyl-CoA-binding protein	-0.62	-2.87	-2.32	-2.46	-1.82	-0.45	0.046	0.004	0.003	0.162	0.262	0.902
Albumin	0.26	1.85	1.62	2.12	1.8	-0.08	0.499	0.063	0.052	0.137	0.173	0.994
Aldo-keto reductase family 1 member B1	-0.28	-2.85	-2.52	-2.65	-2.39	-0.15	0.822	0.005	0.001	0.104	0.080	0.983
Aldo-keto reductase family 1 member C15	-0.28	-2.15	-1.74	-1.7	-1.3	-0.45	0.793	0.063	0.046	0.581	0.594	0.902
Alpha-1-acid glycoprotein	0.86	2.25	1.62	2.37	1.92	-0.33	0.000	0.015	0.051	0.074	0.141	0.902
Alpha-1-antiproteinase	0.42	2.8	2.2	2.87	2.37	-0.04	0.114	0.002	0.004	0.022	0.046	0.994
Alpha-1-inhibitor 3	0.87	2.57	1.69	2.56	1.75	-0.01	0.000	0.004	0.040	0.042	0.190	0.989
Alpha-1-macroglobulin	0.75	2.59	1.78	2.72	1.7	0.04	0.001	0.004	0.028	0.031	0.212	0.977
Alpha-2-HS-glycoprotein	0.46	2.87	2.67	2.68	1.95	0.55	0.076	0.001	0.000	0.033	0.141	0.596
Alpha-2-macroglobulin	0.55	2.7	2.37	3.2	2.63	-0.44	0.021	0.002	0.002	0.007	0.020	0.902
Alpha-actinin-4	-0.74	-0.51	0.23	-6.64	-6.64	6.64	0.008	0.998	0.974	0.000	0.000	0.000
Amine oxidase [flavin-containing] A	-0.75	-0.42	0	-0.93	-0.61	0.65	0.007	0.998	0.993	0.987	0.988	0.484
Angiotensinogen	0.88	2.34	1.46	2.26	1.61	-0.16	0.000	0.010	0.093	0.096	0.252	0.983
Annexin A1	-0.2	1.06	1.61	0.99	1.3	0.11	0.935	0.476	0.054	0.680	0.464	0.940
Apolipoprotein A-I	0.54	2.44	1.78	2.66	1.99	-0.14	0.026	0.007	0.028	0.034	0.129	0.983
Apolipoprotein E	0.55	3.81	3.18	3.66	2.93	0.05	0.022	0.000	0.000	0.001	0.006	0.977
ATP synthase membrane subunit DAPIT, mitochondrial	-0.24	-6.64	-6.64	-0.33	-0.1	-6.64	0.907	0.000	0.000	0.999	0.998	0.000
Band 3 anion transport protein	0.94	1.63	0.76	0.85	-0.02	0.71	0.000	0.125	0.635	0.762	0.998	0.375
Beta-2-glycoprotein 1	0.53	4.1	3.05	4.05	3.1	-0.06	0.027	0.000	0.000	0.000	0.003	0.998
Beta-enolase	-0.61	-2.73	-1.99	-2.14	-1.41	-0.37	0.057	0.007	0.015	0.304	0.529	0.902
C4b-binding protein alpha chain	6.64	6.64	2.59	6.64	1.78	0.5	0.000	0.000	0.000	0.000	0.180	0.664
Carbonic anhydrase 2	0.63	1.25	0.32	0.51	-0.34	0.75	0.006	0.330	0.960	0.987	0.998	0.307
Carbonyl reductase [NADPH] 1	-0.63	-6.64	-6.64	-0.83	-0.38	-6.64	0.041	0.000	0.000	0.999	0.998	0.000
Carboxypeptidase B2		6.64	6.64	6.64	6.64	0.09		0.000	0.000	0.000	0.000	0.954
Ceruloplasmin	0.71	2.66	2.1	2.9	2.09	-0.03	0.001	0.003	0.006	0.020	0.100	0.994
Clusterin	0.2	5.65	5.27	5.33	5.15	0.39	0.659	0.000	0.000	0.000	0.000	0.847

Coactosin-like protein	0.95	2.52	1.57	2.84	1.89	-0.54	0.000	0.005	0.062	0.023	0.143	0.776
Complement C3	0.63	3.21	2.47	3.15	2.4	0.16	0.006	0.000	0.001	0.009	0.044	0.920
Complement C4	0.19	3.17	2.55	2.77	2.28	0.36	0.695	0.000	0.001	0.027	0.058	0.872
Complement component C9	0.29	3.67	3.53	3.51	3.24	0.19	0.422	0.000	0.000	0.002	0.002	0.902
Coronin-1A	1.31	3.23	1.92	3.67	2.36	-0.45	0.000	0.000	0.014	0.001	0.046	0.902
C-reactive protein	0.63	3.53	2.64	3.94	3	-0.69	0.006	0.000	0.000	0.000	0.004	0.567
Creatine kinase M-type	-0.27	-1.94	-1.74	-2.02	-1.84	0.27	0.827	0.123	0.046	0.371	0.252	0.902
Cysteine and glycine-rich protein 3	-0.1	-1.59	-1.72	-2.07	-2.19	0.14	0.985	0.298	0.050	0.348	0.136	0.922
Cytochrome c oxidase subunit 6A1, mitochondrial	1.62	0.25	-1.62	-6.64	-6.64	6.64	0.000	0.998	0.071	0.000	0.000	0.000
Elastin	-0.04	1.43	1.17	-0.07	-0.06	1.69	0.989	0.214	0.249	0.999	0.998	0.000
Elongation factor 1-alpha 1	0.15	2.16	2.03	2.86	2.6	-0.66	0.807	0.021	0.009	0.022	0.022	0.596
ERO1-like protein alpha	-3.83	-6.64	-6.64	-2.94	0.89	-6.64	0.000	0.000	0.000	0.048	0.632	0.000
F-actin-capping protein subunit beta	-0.25	-0.56	-0.38	0.45	0.7	-1.26	0.872	0.998	0.970	0.987	0.811	0.031
Fatty acid-binding protein, heart	-0.11	-2.53	-2.19	-2.77	-2.55	-0.22	0.983	0.016	0.006	0.078	0.050	0.946
Fetuin-B	0.55	2.9	2.26	2.93	2.39	-0.07	0.021	0.001	0.003	0.019	0.045	0.994
Fibrinogen alpha chain	0.79	5.18	4.38	4.8	3.98	0.36	0.000	0.000	0.000	0.000	0.000	0.872
Fibrinogen beta chain	0.86	5.43	4.82	5.05	4.48	0.42	0.000	0.000	0.000	0.000	0.000	0.782
Fibrinogen gamma chain	0.68	5.51	5.01	5.04	4.48	0.44	0.003	0.000	0.000	0.000	0.000	0.776
Fibronectin	0.52	4.34	3.69	4.13	3.49	0.25	0.035	0.000	0.000	0.000	0.000	0.902
Galectin-3	1.57	3.72	2.14	4.44	2.83	-0.73	0.000	0.000	0.005	0.000	0.009	0.505
Gelsolin	0.46	4.24	3.66	4.64	4.2	-0.47	0.072	0.000	0.000	0.000	0.000	0.884
Haptoglobin	0.78	2.81	2.3	3.22	2.5	-0.08	0.000	0.001	0.002	0.007	0.031	0.994
Hemopexin	0.75	2.81	2.2	2.96	2.25	-0.08	0.001	0.001	0.004	0.018	0.062	0.994
Histidine-rich glycoprotein	0.84	2.53	1.7	2.31	1.76	0.09	0.000	0.005	0.039	0.086	0.190	0.954
Histone H1.0	0.08	-2.33	-1.88	-0.66	-0.76	-1.15	0.928	0.046	0.035	0.999	0.892	0.114
Histone H2A type 2-A	-6.64	1.92	6.64	2.83	6.64	-0.9	0.000	0.050	0.000	0.024	0.000	0.267
Hydroxymethylglutaryl-CoA lyase, mitochondrial	-0.07	-6.64	-6.64	-6.64	-6.64		0.989	0.000	0.000	0.000	0.000	
Ig kappa chain C region, A allele	0.62	3.06	2.81	2.71	2.27	-0.21	0.008	0.000	0.000	0.031	0.059	0.954
Inter-alpha-trypsin inhibitor heavy chain H3	0.92	4.02	3.14	3.78	3.01	0.13	0.000	0.000	0.000	0.001	0.004	0.929
Keratin, type I cytoskeletal 13	0.14	-3.09	-3.95	-1.83	-2.61	-1.34	0.827	0.002	0.000	0.508	0.045	0.028
Keratin, type I cytoskeletal 17	-0.64	-0.25	0.46	-0.42	0.29	0.23	0.037	0.998	0.894	0.999	0.998	0.902
Keratin, type I cytoskeletal 42	-0.77	-0.56	-0.23	-0.38	0.3	-0.31	0.006	0.998	0.985	0.999	0.998	0.902

Keratin, type II cytoskeletal 1b	0.28	2.22	2.15	1.46	1.36	0.67	0.454	0.017	0.005	0.468	0.423	0.445
Keratin, type II cytoskeletal 73	0.89	-0.2	-1.23	0.5	-0.56	-0.97	0.000	0.998	0.261	0.987	0.998	0.219
Keratin, type II cytoskeletal 75	-1	-0.65	-0.26	-1.65	-0.41	0.76	0.000	0.994	0.985	0.617	0.998	0.285
Methanethiol oxidase	-0.71	-2.31	-1.64	-1.8	-1.14	-0.51	0.015	0.037	0.066	0.525	0.658	0.872
Mitochondrial fission 1 protein	-0.16	-6.64	-6.64	-1.65	-1.21	-6.64	0.974	0.000	0.000	0.617	0.600	0.000
Murinoglobulin-1	0.82	2.54	1.92	2.77	2.09	-0.16	0.000	0.005	0.014	0.027	0.100	0.983
Myoglobin	-0.12	-2.39	-2.41	-2.93	-2.82	-0.08	0.981	0.028	0.002	0.048	0.022	0.994
Myosin light chain kinase 3	-0.3	-6.64	-6.64	-2.03	-1.83	-6.64	0.741	0.000	0.000	0.367	0.219	0.000
Myosin-11 (Fragments)	-1.27	1.21	2.49	-0.01	-0.34	1.33	0.000	0.416	0.001	0.999	0.998	0.005
Myosin-9	0.07	1.92	1.93	1.87	1.75	0.16	0.955	0.050	0.014	0.222	0.190	0.920
Nucleoside diphosphate kinase B	-0.22	-2.67	-2.15	-1.59	-1.39	-0.51	0.922	0.009	0.007	0.622	0.549	0.858
Osteopontin		6.64	6.64	6.64	6.64	0.26		0.000	0.000	0.000	0.000	0.902
Phosphatidylethanolamine-binding protein 1	-0.22	-2.75	-2.33	-2.69	-2.39	-0.08	0.908	0.007	0.003	0.096	0.080	0.994
Phosphoglycerate mutase 2	-0.34	-2.18	-1.85	-2.32	-2.03	0.14	0.641	0.056	0.030	0.210	0.172	0.922
Plasminogen	0.75	6.11	4.87	6.01	4.88	0.13	0.001	0.000	0.000	0.000	0.000	0.931
Plastin-3	-0.18	3.01	3.13	3.39	3.52	-0.39	0.960	0.001	0.000	0.004	0.000	0.902
Prosaposin	-0.09	2.1	1.93	2.81	2.35	-0.74	0.989	0.027	0.014	0.025	0.047	0.505
Protein S100-A4	2.3	2.67	0.36	3.91	1.34	-0.99	0.000	0.003	0.950	0.000	0.440	0.200
Protein S100-A8	3.58	6.64	2.97	6.51	2.89	0.54	0.000	0.000	0.000	0.000	0.007	0.602
Protein S100-A9	1.52	6.21	4.21	5.46	3.24	0.86	0.000	0.000	0.000	0.000	0.002	0.210
Protein Z-dependent protease inhibitor	6.64	6.64	2.02	6.64	2.02	0.02	0.000	0.000	0.009	0.000	0.122	0.983
Protein-glutamine gamma-glutamyltransferase K	-0.72	-0.44	0.28	-6.64	-6.64	6.64	0.011	0.998	0.970	0.000	0.000	0.000
Prothrombin	0.68	4.66	3.92	4.49	3.75	0.32	0.003	0.000	0.000	0.000	0.000	0.902
Serine protease inhibitor A3K	0.6	2.07	1.44	2.2	1.36	-0.07	0.011	0.030	0.097	0.113	0.423	0.994
Serine protease inhibitor A3L	0.89	2.46	1.67	2.63	1.85	-0.22	0.000	0.006	0.043	0.036	0.151	0.940
Serine protease inhibitor A3M (Fragment)	6.64	6.64	1.19	6.64	1.23	-0.04	0.000	0.000	0.234	0.000	0.518	0.994
Serine protease inhibitor A3N	0.66	2.66	2.22	2.81	2.17	0.1	0.004	0.003	0.003	0.025	0.080	0.948
Serotransferrin	0.61	2.62	2.02	2.52	1.92	0.15	0.009	0.003	0.009	0.048	0.141	0.922
Serum paraoxonase/arylesterase 1	6.64	6.64	1.44	6.64	1.3	0.14	0.000	0.000	0.097	0.000	0.463	0.922
Small muscular protein	-0.52	-1.99	-1.73	-2.02	-1.95	0.19	0.157	0.106	0.048	0.369	0.204	0.902
Superoxide dismutase [Cu-Zn]	-0.52	-2.34	-1.86	-1.92	-1.28	-0.45	0.161	0.033	0.029	0.432	0.598	0.902
T-kininogen 1	0.54	2.86	2.2	2.93	1.91	0.06	0.027	0.001	0.004	0.019	0.141	0.977

T-kininogen 2	0.41	2.4	1.92	2.59	1.92	-0.09	0.129	0.008	0.014	0.039	0.141	0.994
Transgelin	-2.28	1.03	3.32	-3.02	-1.05	4.36	0.000	0.508	0.000	0.039	0.678	0.000
Transketolase	-0.58	2.18	2.36	2.7	2.73	-0.4	0.082	0.020	0.002	0.032	0.013	0.902
Triosephosphate isomerase	-0.4	-2.13	-1.7	-1.99	-1.56	-0.09	0.456	0.066	0.054	0.388	0.443	0.994
Tripartite motif-containing protein 72	-0.13	-1.92	-1.8	-2.42	-2.29	0.43	0.981	0.127	0.037	0.180	0.107	0.776
Tropomyosin alpha-3 chain	0.1	1.8	1.77	1.97	1.85	-0.3	0.908	0.074	0.029	0.192	0.151	0.907
Tropomyosin alpha-4 chain	-0.25	1.45	1.7	1.39	1.57	0.32	0.872	0.207	0.039	0.523	0.281	0.902
Vitamin D-binding protein	0.52	1.8	1.39	2.74	1.94	-0.4	0.034	0.075	0.119	0.030	0.141	0.902

Supporting Table S4. Pathways analyses including the significantly up- or downregulated proteins were performed using EnrichR with Reactome's cell signaling database. The pathways were ranked by the combined score.

Provided as excel document
